# Metagenomics of the Deep Mediterranean, a Warm Bathypelagic Habitat

**DOI:** 10.1371/journal.pone.0000914

**Published:** 2007-09-19

**Authors:** Ana-Belen Martín-Cuadrado, Purificación López-García, Juan-Carlos Alba, David Moreira, Luis Monticelli, Axel Strittmatter, Gerhard Gottschalk, Francisco Rodríguez-Valera

**Affiliations:** 1 División de Microbiología, Universidad Miguel Hernández, San Juan de Alicante, Spain; 2 Unité d'Ecologie, Systématique et Evolution, Centre National de la Recherche Scientifique, Université Paris-Sud, Orsay, France; 3 Istituto per l'Ambiente Marino Costiero, Consiglio Nazionale delle Ricerche, Sezione di Messina, Messina, Italy; 4 Laboratorium für Genomanalyse, Institut für Mikrobiologie und Genetik, Georg-August-University Göttingen, Göttingen, Germany; Centre for DNA Fingerprinting and Diagnostics, India

## Abstract

**Background:**

Metagenomics is emerging as a powerful method to study the function and physiology of the unexplored microbial biosphere, and is causing us to re-evaluate basic precepts of microbial ecology and evolution. Most marine metagenomic analyses have been nearly exclusively devoted to photic waters.

**Methodology/Principal Findings:**

We constructed a metagenomic fosmid library from 3,000 m-deep Mediterranean plankton, which is much warmer (∼14°C) than waters of similar depth in open oceans (∼2°C). We analyzed the library both by phylogenetic screening based on 16S rRNA gene amplification from clone pools and by sequencing both insert extremities of *ca.* 5,000 fosmids. Genome recruitment strategies showed that the majority of high scoring pairs corresponded to genomes from Rhizobiales within the Alphaproteobacteria, *Cenarchaeum symbiosum*, Planctomycetes, Acidobacteria, Chloroflexi and Gammaproteobacteria. We have found a community structure similar to that found in the aphotic zone of the Pacific. However, the similarities were significantly higher to the mesopelagic (500–700 m deep) in the Pacific than to the single 4000 m deep sample studied at this location. Metabolic genes were mostly related to catabolism, transport and degradation of complex organic molecules, in agreement with a prevalent heterotrophic lifestyle for deep-sea microbes. However, we observed a high percentage of genes encoding dehydrogenases and, among them, *cox* genes, suggesting that aerobic carbon monoxide oxidation may be important in the deep ocean as an additional energy source.

**Conclusions/Significance:**

The comparison of metagenomic libraries from the deep Mediterranean and the Pacific ALOHA water column showed that bathypelagic Mediterranean communities resemble more mesopelagic communities in the Pacific, and suggests that, in the absence of light, temperature is a major stratifying factor in the oceanic water column, overriding pressure at least over 4000 m deep. Several chemolithotrophic metabolic pathways could supplement organic matter degradation in this most depleted habitat.

## Introduction

The deep ocean is one of the most important and less understood microbial-driven ecosystems on Earth. Since the recognition of the essential role of microbes on the ocean water column [1], most marine microbiology studies have been devoted to the photic zone, where microbial cell density and activity are high and most primary production occurs. Microbial communities in deeper oceanic layers, particularly below 1,000 m (bathypelagic and abyssal waters), have low cell densities and low metabolic activities partially due to the extreme reigning conditions. Not only light is absent but deep waters are most often oligotrophic, pressure increases and temperature decreases very rapidly to reach average values around 2°C in the open ocean. Despite so, given the vast dimensions of the deep ocean, occupying nearly two thirds of the planet's surface and reaching an average depth of 3,800 m, the microbial community of this ecosystem becomes fundamental for global biogeochemical cycling. Studying the microbial communities in offshore deep marine locations has always been difficult and demanding. Pure culture approaches are very difficult to apply, and relevant (not opportunistic) microbes are extremely difficult to isolate. Molecular approaches based on the amplification of small subunit ribosomal RNA genes improved the situation markedly [2]–[4], but still leave the deep water mass of most oceans under-sampled and provide no functional information about lineages that rarely have close cultured relatives. Metagenomics, the study of genetic and genomic information from whole environmental communities, has brought some hope to get insights about the metabolic potential and evolutionary history of uncultured marine microbes, thus sidestepping the need for culturing or isolation [5]–[7]. Except for a few small-scale analyses of genome fragments from archaea and bacteria from mesopelagic waters [8]–[10], metagenomic studies in the ocean have been also primarily devoted to surface waters (e.g. [11], [12], including recent large-scale comparative studies along surface transects [13]. So far, the only large-scale metagenomic analysis of deep-sea communities correspond to a comparative study that DeLong and co-workers carried out at different depths in the water column at the North-Pacific Subtropical Gyre ALOHA station, ranging from 10 to 4,000 m depth [14]. Extending metagenomic analyses to other deep-sea communities would help unravel important questions about metabolism and lifestyle of deep-sea microbes. For instance, although the deep ocean is generally considered a metabolic sink for the organic matter produced in the photic zone, autotrophic archaea (crenarchaeota), possibly ammonia-oxidizers [15], [16], are mostly abundant in deep waters [17].

Pressure is thought to have a significant influence in deep-sea stratification, as piezophilic (barophilic) species have been isolated [18], and specific adaptations such as pressure regulated operons are present in some deep-sea bacteria [19], [20]. However, along with high pressure, low temperatures also characterize deep-sea waters. They limit growth rate through its slowing-down effect on metabolic chemical reactions and, consequently, psycrophilic organisms develop particular adaptations tending to increase protein flexibility and reactivity [21]. A few exceptional locations provide the chance to study microbial communities at high oceanic depth without being affected by near zero temperatures, therefore providing the opportunity to assess the relative importance of pressure and temperature in microbial adaptation at genomic level in the natural environment. The largest and most ecologically relevant is the Mediterranean Sea. Although it has an exceptionally deep basin for a basically landlocked sea, reaching 5,000 m at its deepest Eastern end and with an average depth of 2,000 m, the Mediterranean is free of cold polar water that cannot get over the sill of the Gibraltar Strait [22]. The deep Mediterranean water mass never gets below 13.5°C, providing a model for a deep relatively warm bathypelagic habitat. The Ionian Sea at the South East of Sicily possesses very pristine and stable deep waters. On this ground, the Ionian station Km3 has been extensively studied as a candidate site for a neutrino telescope (NEMO) (http://nemoweb.lns.infn.it/publication.htm). The prokaryotic diversity of a 3,000 m deep sample from the station Km3 was studied recently by analyzing 16S rRNA gene libraries, which revealed a wide variety of prokaryotic lineages [4]. This prompted us to construct a metagenomic fosmid library from the same sample.

Here we report the construction and analysis of such a fosmid metagenomic library from 3,000 m deep Km3 small plankton (0.2–5 µm fraction). The gene repertoire identified supports a predominant heterotrophic lifestyle for this deep Mediterranean community and suggests versatility in energy-gaining mechanisms, including a widespread use of CO oxidation.

## Results

The Km3 metagenomic library was constructed from planktonic fractions smaller than 5 µm of seawater collected at 3,010 m depth that was at a temperature of nearly 14°C and 38.7% salinity (see Methods). It contained *ca.* 20,000 fosmid clones, from which approximately one-fourth was subjected to bi-directional end sequencing, yielding 7.2 Mbp of DNA sequence from the approximately 725 Mbp total archive (Table 1). This represents raw sequence of approximately 2 prokaryotic genome equivalents (considering an average genome size of 3.5 Mbp). Consistently with this estimation we retrieved a single hit to *recA* [23] a gene that has been used to establish the number of genome (or cell) equivalents in a metagenomic library [23].

**Table 1:** General features of the Km3 metagenome.

Major feature	Subcategory	Value
Number of fosmid clones		20,767
Total archived sequence		725 Mbp^a^
Number of 16S rRNA genes amplified by PCR	Archaeal	28
	Bacterial b	16
Number of high-quality fosmid-end sequences		9,048
Average length of sequence reads		794 bp
Total generated sequence length		7,184 kbp
Average GC content		50.1%
Fosmid-end sequences	belonging to defined taxonomic groups (<1e-50)	23.4%
	of ambiguous taxonomic ascription (1e-7 to 1e-50)	53.7%
	with homologues only in GOS^c^	11.4%
	without homologues in any database	11.4%
Distribution of sequences in major COG categories		
	Metabolism	50.4%
	Information storage and processing	17.1%
	Cellular Processes and Signaling	16.0%
	Poorly characterized	16.5%
Distribution of sequences in major KEGG categories		
	Metabolism	70.6%
	Genetic Information Processing	17.4%
	Environmental Information Processes	10.2%
	Cellular Processes	1.7%

BLAST cut-off values used in each case are shown in brackets.

a Considering average insert sizes of 35 kbp

b Only fosmids containing bacterial ITS regions different in size of that from *E. coli* were identified.

c GOS, Global Ocean Surveyor database {Rusch, 2007 #16}

### Community composition

**Figure 1:**
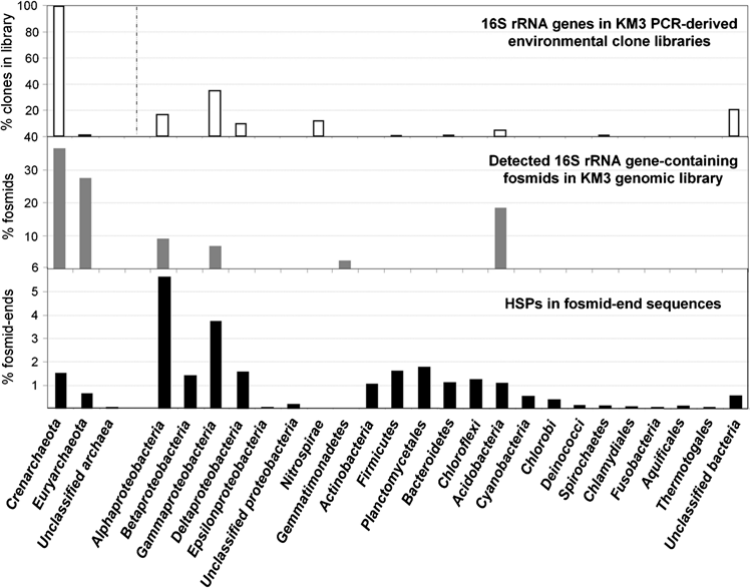
Prokaryotic taxa identified in Km3 3,000 m-deep plankton inferred from fosmid-ends and 16S rRNA detection in environmental and metagenomic libraries. Relative abundances of PCR-amplified 16S rRNA genes in environmental libraries are from Ref.4. For bacteria in the Km3 metagenomic library (central panel), only 16S rRNA genes whose adjacent ITS can be distinguished in size from that of *E. coli* were detected.

**Figure 2:**
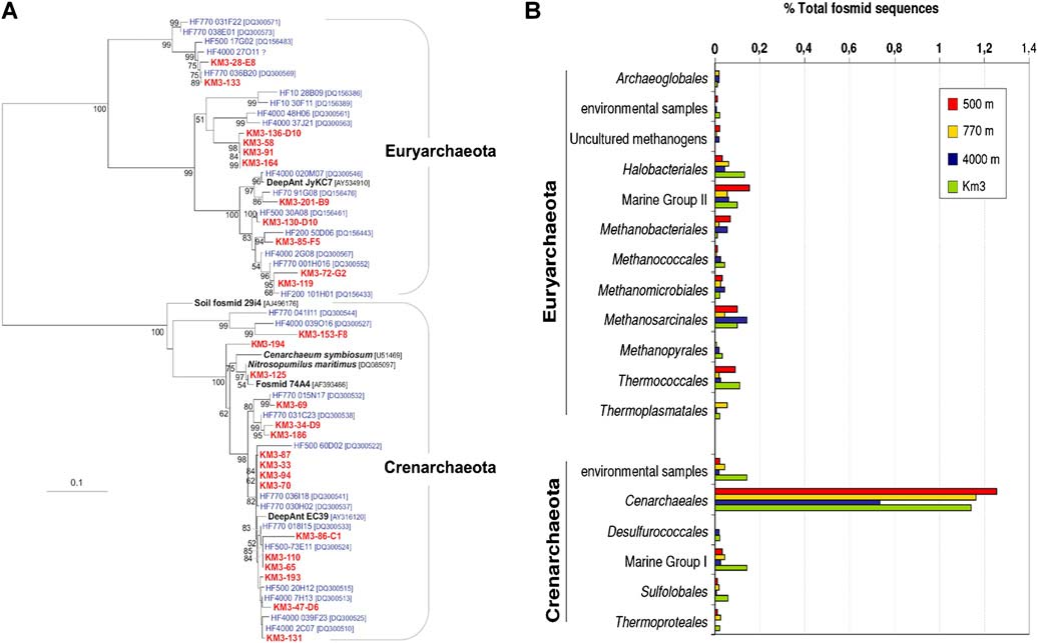
Taxonomy of archaeal fosmids. A, Phylogenetic tree of 16S rRNA genes amplified from the metagenomic library Km3. The tree was constructed by maximum likelihood using PhyML and a total of 704 non ambiguously aligned positions. Non-parametric bootstrapping was performed upon 1,000 replicates. Only bootstrap values above 50 are shown. Km3 and ALOHA water column sequences are indicated in red and blue, respectively. B, Comparative taxonomic distribution obtained by best BLAST hit (see methods) of archaeal fosmid-ends in KM3 and ALOHA deep-sea libraries [14]. Marine group I and environmental samples are non-taxonomic designations as used in databases.

The analysis of metagenomic libraries can complement diversity studies based on 16S rRNA gene PCR amplification, since they are not subjected to the same biases. We used a double approach to estimate the prokaryotic diversity in the Km3 library, PCR amplification of 16S rRNA genes in pooled clones of the whole library, and phylogenetic assignment of fosmid insert terminal sequences from approximately 5,000 clones. In the case of archaea, we used different primer combinations to recover a maximal variety of archaeal genes. We detected a total of 28 archaeal fosmid containing 16S rRNA genes (Table 1 and Fig. 1). Eighteen out of the 28 archaeal clones were crenarchaeota, most of them members of the *bona fide* marine Group I crenarchaeota, and one of them belonging to the recently identified pSL12-related cluster or group 1A [14], [24]. The remaining 11 clones belonged to the Euryarchaeota of the marine Group II (9 clones) and Group III (2 clones) (Fig. 2A). A similar trend could be observed from fosmid-end phylogenetic ascription (Fig. 1). We applied expectation cut-off values of 1e-50 for binning our fosmid sequences within known taxa with sequences in databases; approximately 23% (Table 1) fulfilled this condition. Although this proportion might seem limited, these sequences represent a random sample from the total diversity, hence providing a reasonably proxy to the prokaryotic census in the deep Mediterranean. Based on fosmid-ends, about 9% of the microbial diversity in Km3 would be archaea, which is in the same order of magnitude that the 15% estimated by archaeal 16S rRNA gene-containing fosmids in the metagenomic library. Likewise, crenarchaeota were also found in equivalent relative proportions (∼60%) compared to euryarchaeota (∼40%) in the metagenomic library (Fig. 1). Our results confirm and extend previous studies showing that crenarchaeota increase their relative abundance at high depth [14], [25], although euryarchaeota still keep significant levels (see Fig.S5 in Ref.14 and Fig. 2B).

**Figure 3:**
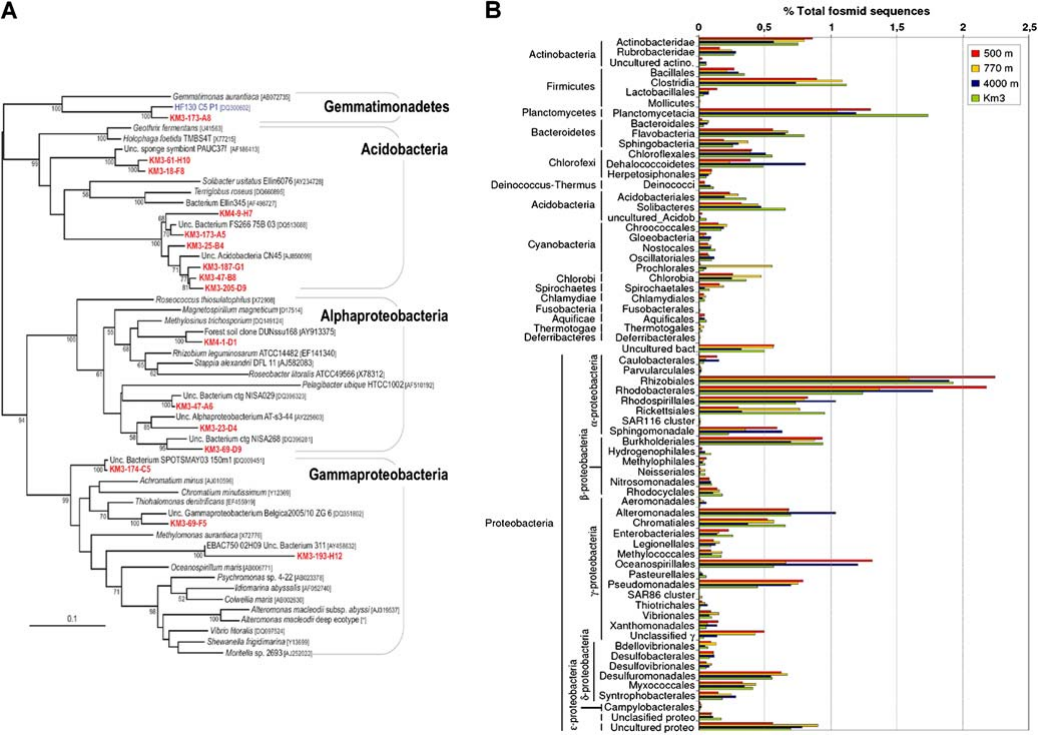
Taxonomy of bacterial fosmids. A, Phylogenetic tree of 16S rRNA genes amplified from the metagenomic library Km3 whose adjacent intergenic spacers differ in size from those of *Escherichia coli*. The tree was constructed by maximum likelihood using PhyML and a total of 1128 non ambiguously aligned positions. Non-parametric bootstrapping was performed upon 1,000 replicates. Only bootstrap values above 50 are shown. Km3 and ALOHA water column sequences are indicated in red and blue, respectively. Unc., uncultured. [*], AA0D00000000 genome sequence underway. B, Comparative taxonomic distribution of bacterial fosmid-ends by best BLAST hits in KM3 and ALOHA deep-sea libraries [14].

According to fosmid-end taxon-binning, the bacterial component in the Km3 metagenomic library was dominated by the Proteobacteria, and within them, by the Alphaproteobacteria, followed by Gamma-, Delta- and Betaproteobacteria (Fig. 1). Gram positive bacteria, both Actinobacteria and Firmicutes were also relatively abundant. There might be a slight bias in the proportion of these phyla due to the fact that, in spite of an increasing effort to widen the taxonomic spectra of sequenced genomes, there are many more genomes available from proteobacteria and Gram positive bacteria than from other taxa, which might result in a slight overrepresentation of these lineages. However, other taxonomic groups appeared also very abundant, namely Planctomycetales, Chloroflexi, Bacteroidetes and Acidobacteria (Fig. 1). The presence of Acidobacteria was also patent by amplification of 16S rRNA genes from pooled clones. Acidobacterial 16S rRNA gene sequences were also detected in deep sea waters in DeLong et al.'s study [14]. Even with the highly limited approach used here to detect bacterial ribosomal operons by PCR (see methods), 8 out of 18 16S rRNA genes detected here belonged to Acidobacteria (Fig. 3A) Similarly, although we detected Gemmatimonadetes 16S rRNA genes in the Km3 metagenomic library, fosmid-end sequences corresponding to this group could not be recognized in the absence of available complete genome sequences. Sequences belonging to groups for which representative genome sequences are still missing may fall in the category of unknown proteins (Table 1) if they contain so far non-described proteins, which appear to be numerous according to recent massive sequence analyses [26]. Proteins with known homologues may also be of ambiguous taxonomic classification or artificially placed in another phylum with relatively low scores as a result of insufficiently close relative genomes in databases. Roughly 50% of the fosmid-end sequences were included in this category (Table 1). Consequently, the description of the prokaryotic diversity by this approach needs to be taken with caution. Despite so, the diversity pattern observed in deep-sea Km3 waters by this approach does not differ significantly from that observed in the deep ALOHA water column, being especially similar to the 770 m depth sample (Fig. 3B and Ref.14).

### Prevailing organismal genomes

The large sequencing effort of prokaryotic genomes, many of them marine, carried out during the last few years allows a direct comparison of marine metagenomic sequences with complete genomes with a reasonable chance of meaningful results. BLASTX HSPs with scores smaller than 1e-50 allowed assigning with confidence fosmid-end sequences to a taxonomic category containing the hit genome. Genomes recovering at least 10 high-score Km3 hits are indicated in Table 2.

**Table 2:** Hallmark genomes recruited by Km3 fosmid-end sequences.

Taxonomic group	Species genome	Number of hits*	% identity	%GC genome	%GC Km3 seq.	O_2_ requirement	Habitat	Metabolism
Alphaproteobacteria Rhizobiales	Rhizobiales (19 genomes)	112	56.53	_	_	aerobic	soil	heterotrophy
	*Rhodopseudomonas* sp. (5 genomes)	27	60.54	_	_	aerobic		
	*Parvibaculum lavamentivorans* DS-1	23	56.31	62.3	53.08	aerobic		
Crenarchaeota	*Cenarchaeum symbiosum*	102	64.47	57.4	36.06	aerobic	marine	autotrophy?
Alphaproteobacteria Rickettsiales	*Pelagibacter ubique* (2 genomes)	84	69.81	29.8/29.7	34.16/33.64	aerobic	marine	oligotrophy
Planctomycetales	*Blastopirellula marina* DSM 3645	78	57.23	57	58.71	aerobic	marine	oligotrophy
Planctomycetales	*Rhodopirellula baltica* SH 1	58	56.65	55.4	58.22	aerobic	marine	heterotrophy
Acidobacteria	*Solibacter usitatus* Ellin6076	56	55.67	61.9	54.64	aerobic	soil	oligotrophy
Chloroflexi	*Dehalococcoides* sp. (3 genomes)	42	56.58	_	_	facultative an.		chemolithotrophy
Alphaproteobacteria Rhodospirillales	*Magnetospirillum* (2 genomes)	36	61.81	65.1/66.4	55.44/56.88	aerobic		heterotrophy
Gammaproteobacteria Pseudomonadales	*Pseudomonas* sp. (13 genomes)	36	58.56	_	_	aerobic		heterotrophy
Alphaproteobacteria Rhodobacterales	*Roseovarius* (3 genomes)	33	61.31	_	_	aerobic		heterotrophy
Acidobacteria	Bacterium Ellin345	32	57.59	58.4	52.43	aerobic	soil	oligotrophy
Betaproteobacteria Burkholderiales	*Burkholderia* sp. (13 genomes)	32	52.33	_	_	aerobic		heterotrophy
Chloroflexi	*Roseiflexus* sp. RS-1	26	52.86	60.4	50.61	facultative an.		photoautotrophy
Deltaproteobacteria Desulfuromonadales	*Geobacter* sp. (4 genomes)	25	55.73	_	_	anaerobic	soil / sediment	chemolitotrophy
Actinobacteria	*Rubrobacter xylanophilus* DSM 9941	23	56.54	70.5	53.21	aerobic	soil	heterotrophy
Gammaproteobacteria	*gamma proteobacterium* KT 71	21	59.61		48.61	aerobic	marine	oligotrophy
Gammaproteobacteria Chromatiales	*Alkalilimnicola ehrlichei* MLHE-1	19	62.71	67.5	52.94	anaerobic		chemolithotrophy
Bacteroidetes Sphingobacteria	*Salinibacter ruber* DSM 13855	18	51.55	66.1	44.88	aerobic	hypersaline	heterotrophy
Gammaproteobacteria Oceanospirillales	*Alcanivorax borkumensis* SK2	16	67.65	54.7	52.68	aerobic		heterotrophy
Gammaproteobacteria Chromatiales	*Nitrosococcus oceani* ATCC 19707	16	62.2	50.3	50.25	aerobic	marine	chemolithotrophy
Betaproteobacteria Burkholderiales	*Ralstonia* sp. (5 genomes)	16	62.11	_	_	aerobic	soil	heterotrophy
Firmicutes Clostridia	*Carboxydothermus hydrogenoformans* Z-2901	16	53.94	42	51.88	facultative an.		chemolithotrophy
Alphaproteobacteria Rhodospirillales	*Rhodospirillum rubrum* ATCC 11170	15	64.42	65.4	55.33	facultative an.		
Gammaproteobacteria Methylococcales	*Methylococcus capsulatus* str. Bath	15	62.49	63.6	52	aerobic		methylotrophy
Deltaproteobacteria Myxococcales	*Anaeromyxobacter dehalogenans 2CP-C*	15	57	74.9	56.86	facultative an.	soil / sediments	heterotrophy
Gammaproteobacteria Alteromonadales	*Marinobacter aquaeolei* VT8	13	62.63	56.9	48.38	aerobic	marine	heterotrophy
Bacteroidetes Flavobacteria	*Flavobacteriales bacterium* HTCC2170	13	59.93	37	48.23	aerobic	marine	heterotrophy
Gammaproteobacteria Chromatiales	*Nitrococcus mobilis* Nb-231	13	57.72	60	51	aerobic		chemolithotrophy
Actinobacteria	*Streptomyces* sp. (4 genomes)	13	55.84	_	_	aerobic	soil	heterotrophy
Gammaproteobacteria	*marine gamma proteobacterium* HTCC2207	12	60.98	49.4	49.25	aerobic	marine	heterotrophy
Cyanobacteria Chroococcales	*Synechococcus* sp. (5 genomes)	11	61.28	_	_			photoautotrophy
Deltaproteobacteria	*delta proteobacterium* MLMS-1	11	58.1	60.1	52.54	anaerobic		chemolithotrophy
Gammaproteobacteria Alteromonadales	*Alteromonas macleodii* ‘Deep ecotype’	10	69.33	44.9	42.3	aerobic	marine	heterotrophy
Betaproteobacteria Burkholderiales	*Bordetella* sp. (5 genomes)	10	51.66	_	_	aerobic		heterotrophy

* BLASTX cut off value1e-50

**Figure 4:**
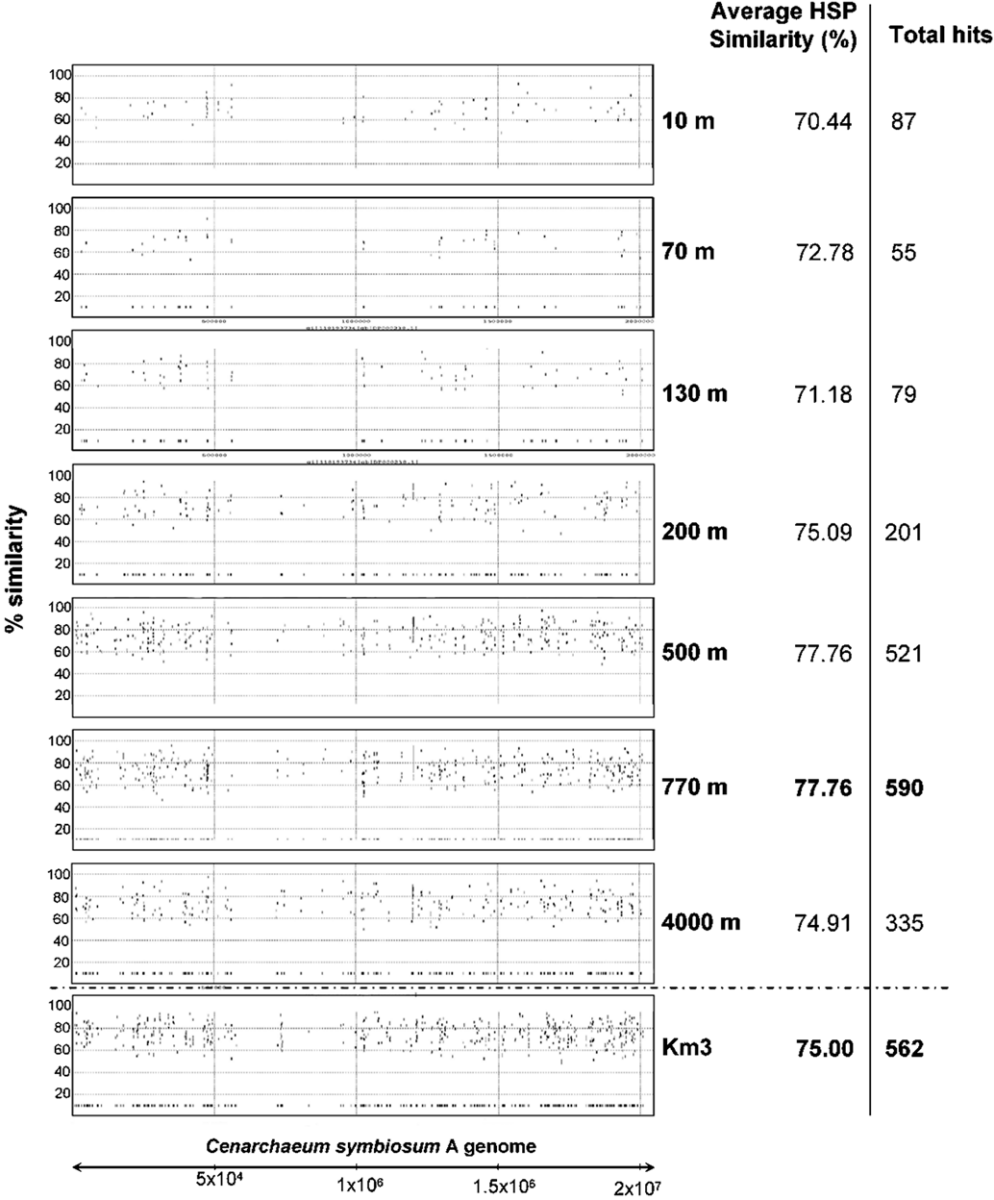
Genome recruitment by *Cenarchaeum symbiosum* A. Individual fosmid-end sequences were aligned to the sequenced strain genome and the alignment-sequence conservation visualized in the form of percent identity plot. Each dot of the graph represents an individual fosmid-end sequence aligned along its homologous region in *C. symbiosum* A genome. *Y* axis reflects its nucleotide percent identity to the syntenic region. Both Km3 and ALOHA water column datasets were used.

The most striking observation was the consistent recruitment of alphaproteobacterial genomes, particularly from Rhizobiales, which accounted for the largest number of hits considered collectively. Also within the Alphaproteobacteria, the two strains of *Pelagibacter ubique* (84 hits) were among the most frequently matched by our sequences, in agreement with the highly recorded prevalence in oceans [27]. Genomes from other Proteobacteria (Gammaproteobacteria, followed by Beta- and Deltaproteobacteria) were also, though to a much lesser extent, well represented. Similarly, the genome of the archaeon *Cenarchaeum symbiosum* A recruited a large number of hits, supporting a relative high proportion of related crenarchaeota in the deep-sea (Fig. 4). The lack of euryarchaeotal Group II and III genomes prevents a similar comparison, although the relative high number of hits against the few available small environmental Group II genome fragments advances a similar situation to that of Group I crenarchaeota and the *C. symbiosum* genome (Fig. 2B). Planctomycetes followed in relative abundance, with *Blastopirellula marina* and *Rhodopirellula baltica* as frequently hit genomes, confirming the dominant role played by these organisms in most open ocean oligotrophic waters [28]. Candidatus *Kuenenia stuttgartiensis,* which lives in extremely organic-rich environments oxidizing ammonium anaerobically [29] receives also some genome hits (Table 2). Such anammox bacteria could live associated to sinking particles with anoxic niches. If this is confirmed, it would imply that ammonium oxidation, either aerobic or anaerobic, is a very important process in the deep ocean, and key to the nitrogen cycle. Less predictable but also supported by recent metagenomic and 16S rRNA-based studies is the abundance of Acidobacteria, represented by the genomes of *Solibacter usitatus* Ellin 6077 and the Bacterium Ellin345. Chloroflexi were also abundantly represented through the genomes of *Roseiflexus* RS1 and *Dehalococcoides ethenogenes.* The concordance of the two end hits with the expected distance found in the corresponding genome was only found in few cases, among them is that of *Alteromonas macleodii* DE, an isolate obtained from a location not very distant from the sampling site [30], *P. ubique* HTCC 1062, *Cellulophaga sp.* MED134 and *Magnetospirillum magnetotacticum MS-1.*

Among the organisms whose genomes had more HSPs with Km3 sequences, aerobic heterotrophic metabolism seems to prevail as a lifestyle, but additional patterns arise. Many retrieved genomes correspond to marine oligotrophs or to bacteria having diverse degradative potential including xenobiotics and/or recalcitrant organic compounds and polymers. This would tend to support a dominant role for heterotrophy, and particularly the degradation of complex organic molecules. The relatively large number of hits to some bacterial taxa seems particularly remarkable, for example the Rhizobiales. These are typical soil inhabitants that may perform symbiotic nitrogen fixation in association with some plants. Accordingly, one of the genes identified is involved in rhizopine catabolism (42% similarity to *mocD*). Rhizopine is produced by some rhizobia and provides a competitive advantage in nodulating symbioses, perhaps as a carbon and nitrogen storage resource [31]. Genes involved in rhizopine degradation were also identified in rhizobia that lack nodulation genes and apparent symbiotic behavior [32]. Similarly, Acidobacteria, another taxonomic group widely distributed in soils, appears fairly abundant. The possibility that Acidobacteria and Rhizobiales are contaminants from the bottom sediment is unlikely, as water samples were collected more than 200 m above the sea floor. Interestingly, Acidobacteria and rhizobia have exchanged genes by horizontal gene transfer as revealed by soil metagenomic analysis [33], suggesting that they might entertain some kind of interaction in habitats where they co-exist. The observation of high abundances of Rhizobiales and Acidobacteria in the deep Mediterranean extends knowledge about the natural habitats of both bacterial groups.

### Gene content and metabolic potential

**Figure 5:**
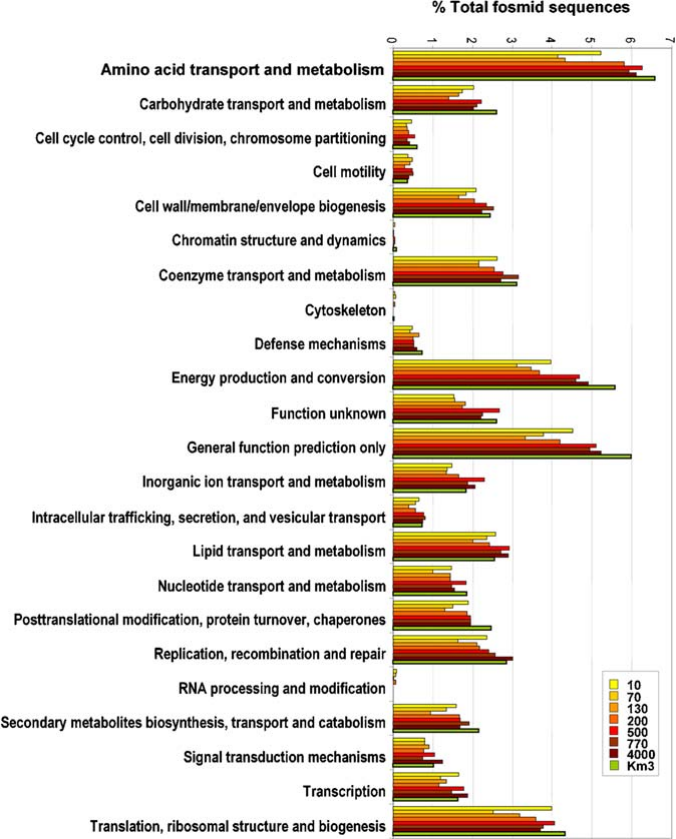
Comparison of COG distribution of fosmid-ends in Km3 and ALOHA water column. Fosmid-ends were classified according to the COG database both Km3 and ALOHA [14] datasets were analyzed (see methods).

**Figure 6:**
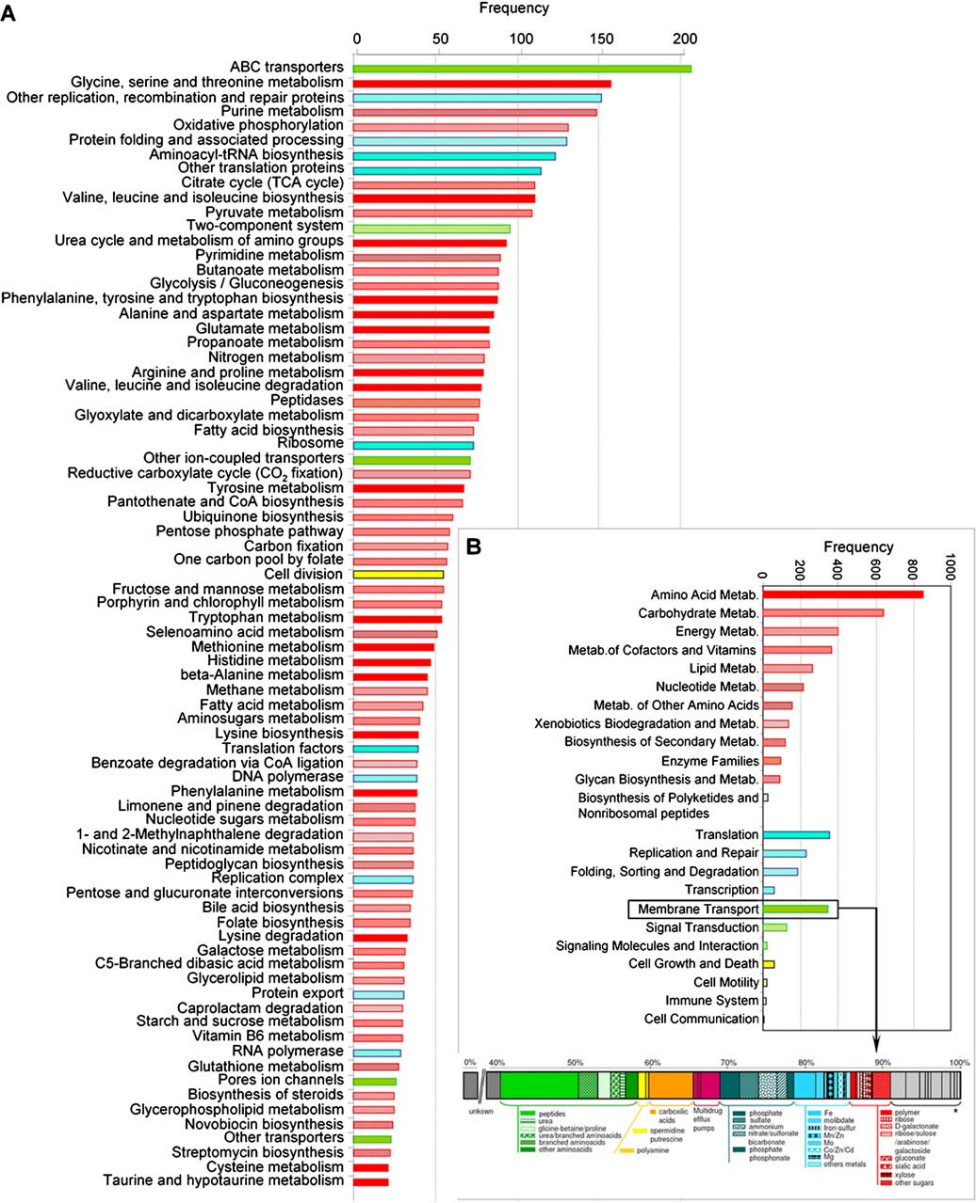
Distribution of Km3 fosmid-ends in KEGG categories. A, Detailed KEGG categories. B, Major KEGG categories and classification by type of substrate of Km3 fosmid-ends identified as transporters. * Other transporters.

We classified Km3 fosmid-end identified ORFs in functional classes according to the Cluster of Orthologous Groups (COG) database [34] and the Kyoto Encyclopedia of Genes and Genomes (KEGG) (http://www.genome.jp/kegg/). The metabolic potential of microorganisms thriving in the deep Mediterranean can be assessed to some extent from the type of metabolic genes encountered. Most of the genes, nearly 50% to 70% according to the COG or the KEGG classification, respectively, were related to metabolism and transport, whereas only 17% corresponded to housekeeping genes involved in information-related processes (Table 1 and 3). The most abundant functional family was that of transporters, and within it, amino acid transporters (Figs. 5 and 6). Transporter systems can tell about nutrient pools or substrates that are present in the environment and that the organisms use. The largest group of transporters in Km3 consisted of the multi-subunit ABC family (64,4% of the identified transporters) and the most represented of this family were transporters for dipeptides/oligopeptides and branched chain amino acids (11,2% and 4.3% sequences, respectively). TRAP transporters, which allow substrate accumulation using an electrochemical ion gradient rather that ATP hydrolysis, were also frequently encountered as well as transporters for carboxylic acids (6.5%), while only 5.7% were sugar/polymer transporters (Fig. 6). The relative high number of peptide and branched chain amino acid transporters suggests that proteins, perhaps associated with sinking marine snow, are an important carbon source for deep-sea microbes. By contrast to shallow waters, with sugars forming an easily accessible labile pool of organic matter, recalcitrant forms of dissolved organic carbon to biological degradation such amides predominate in deep waters [35].

**Table 3:** Hallmark proteins in the Km3 metagenome compared to the deep ALOHA water column.

BLASTX cut-off value	Protein / Protein class	Number of gene/protein per Mbp			
		500	770	4000	Km3
1e-50	RecA	0.3	0.3	0.3	0.1
	DnaK	0.5	0.7	0.6	1.5
	RpoB	0.3	0.3	0.3	0.7
	Pgm	0.0	0.2	0.1	0.4
	PycA	0.5	0.6	1.0	0.7
	GyrB	0.4	0.7	0.7	0.5
	Mdh	1.1	1.0	1.0	1.1
1e-20	Dehydrogenases	40.3	38.1	43.7	59.7
	Carbon-monoxide-dehydrogenase (*cox*)	1.0	1.7	1.0	2.5
	Luciferase-like genes	0.4	1.4	1.5	2.9
	Transposase	1.6	1.7	8.1	4.6
	Phage-related	2.0	1.3	2.5	3.6
	Chaperones	2.1	2.4	2.5	3.7
	Dehalogenases	0.5	0.2	0.6	1.4

Approximately 10% of the identifiable Km3 genes were related to energy production and conversion (Figs. 5 and 6). The best represented pathways were oxidative phosphorylation (131 hits) followed by carbon fixation pathways (129 hits) and nitrogen metabolism (80 hits). In agreement with the deep origin of the sample, genes related to photosynthesis were minoritary. By contrast, genes involved in the degradation of different kinds of compounds, including notably biopolymers and xenobiotics, and catabolic pathways in general were fairly abundant. Among them were several acetone decarboxylases and dehalogenases and KEGG-pathways associated with the degradation of limonene and pinene, 1-and 2-methylnaphthalene, glycosaminoglycan, benzoate, 1,2-dichloroethane, nitrobenzene, gamma-hexachlorocyclohexane, ethylbenzene and fluorene. This reinforces the idea that microorganisms living at this depth are adapted to degrade recalcitrant pools of organic matter. Similarly to previous observations in the ALOHA water column, the enrichment of genes related to pilus, polysaccharide and antibiotic synthesis genes observed might suggest a potential role for a surface-attached lifestyle. Among enzyme-coding genes, oxidases, reductases and oxidoreductases were relatively numerous, followed by carboxylases and decarboxylases. However, by far, the most abundant enzymatic class was that of the dehydrogenases (Table 3). Notably, among the most represented genes of this class were those encoding the different subunits (CoxL, CoxM, CoxS) of carbon monoxide dehydrogenase (CODH) (Table 3). We detected confidently (1e-50) up to 5 *coxL* genes, and this, for about two genome equivalents of Km3 sequence. In addition, at least 10 additional *coxL* sequences were detected by phylogenetic analyses from putative *cox* genes with BLASTX scores >1e-50 (data not shown). In the Sargasso Sea metagenome there was only one *coxL* per 11 genome equivalents [12]. *coxL* was more abundant in aphotic waters in the ALOHA water column, ranging from 1 to 5 copies in the photic region, and from 7 to 10 copies in the aphotic waters column for an average of 3–4 genome equivalents at each depth [14]. The capacity to oxidize CO aerobically without a direct link to autotrophy has been recently identified in several bacteria. For instance, *Silicibacter pomeroyi,* a marine bacterium of the *Roseobacter* clade whose genome has been sequenced [23] possess these genes but lacks autotrophic carbon fixation pathways. Its strategy consists of supplementing heterotrophy with the use of inorganic compounds (CO and sulfide). The capacity to use simultaneously CO and organic substrates is known for several other bacteria, including marine genera such as *Stappia* [36]. Most interestingly, *cox* genes are also present in the genome sequences of the acidobacteria *Solibacter usitatus* and the Acidobacteria Bacterium Ellin345 [37]. Phylogenetic analyses of Km3 *coxL* showed that some of them were clearly related to Alphaproteobacteria, Actinobacteria and Chloroflexi homologues, but not to *Solibacter coxL* (data not shown). Nonetheless, since Km3 Acidobacteria were very diverse (Fig. 3A), it might be possible that some of the phylogenetically unclassified Km3 *coxL* belong to this phylum.

Other genes in relative high numbers in the Km3 metagenome were luciferase-like genes (Table 3), which likely encode monooxygenases. However, it is difficult to advance a function for most of them, since only a few were clearly related to *luxA* (encoding the luciferase alpha subunit and therefore directly involved in bioluminescence).

### Metagenomic comparison of deep Mediterranean samples and the water column at ALOHA station

**Figure 7:**
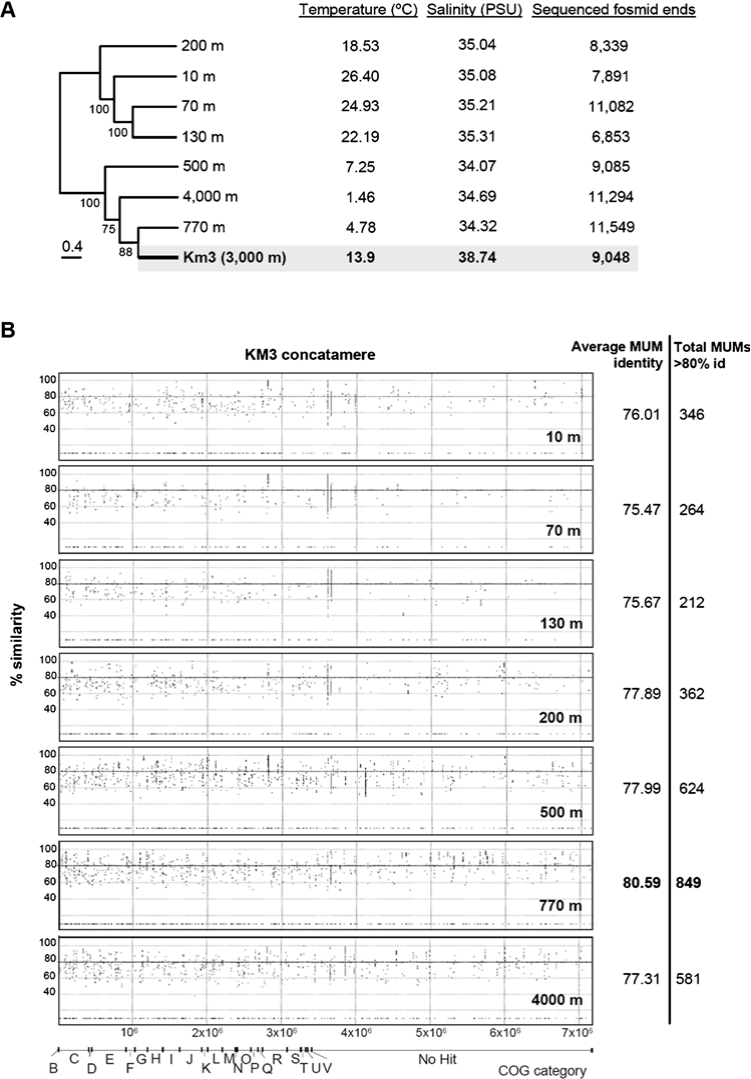
Normalized metagenome comparison of 3000 m-deep Mediterranean Km3 and Pacific ALOHA water column. For normalization, a total of 6,853 sequences (size of the smallest library compared, ALOHA 130 m) from each library were randomly selected and compared. A, Neighbour joining analysis of fosmid-end sequences in Km3 and different depths in ALOHA. Temperature, salinity, and the total number of sequences available for each library are shown on the right; Jackknife values, at nodes. B, Normalized MUMmer plots showing the number of maximal unique matches (MUMs) shared by the 3000 m deep Km3 and the different ALOHA metagenomic libraries. MUMs are distributed as a function of their identity (ordinates) and the type of COG to which they belong (abscises). Average identity values are indicated for each pair of libraries compared. The number of MUMs having more that 80% identity are given to the right of each panel.

The availability of metagenomic sequences from different depths at the Pacific ALOHA station makes it possible to test whether warmer Mediterranean temperatures at similar high depth (3,000–4,000 m) can affect community structure significantly. Of course other environmental parameters, such as salinity or biogeography may affect it as well. However, despite slight differences between the aphotic ALOHA and the deep Km3 metagenomic libraries, the equivalent sequence volume produced per library together with the overall similar general patterns of taxa found (Figs. 2 and 3), gene content (Figs. 5 and 6) and aminoacid usage (data not shown) allows a reasonable comparison between them. We made TBLASTX searches of our Km3 sequences against each one of the depth-related metagenomic libraries in the ALOHA water column to construct a similarity matrix that was analyzed by neighbour-joining (Fig. 7A). The sequence datasets were additionally used to construct coverage maximal unique matches (MUMs) plots (Fig. 7B). The 3,000 m deep Km3 metagenome recruited most hits from the ALOHA aphotic water column, as expected. Surprisingly, contrary to the initial expectation of a higher similarity with the 4,000 m deep sample, enduring analogous pressures, the most abundant and highest MUMs were observed with intermediate deep-water libraries, particularly that of 770 m. This relationship was not limited to sequence similarity, but also gene types (metabolism and cell physiology) and even the frequency of mobile elements (IS, phage related, integrons) in the Mediterranean 3,000 m-deep sample seemed more alike to the upper section of the aphotic zone in the Pacific. We interpret this result as being essentially the consequence of the warmer temperature (13.9°C) of deep Mediterranean waters. Most other biologically relevant parameters are remarkably similar (Table 4). As a matter of fact, although much deeper, the Km3 sample has in common with the ALOHA 500 and 770 m deep libraries intermediate temperatures, 4.8 to 7.2°C, instead of the 1.4°C characterizing the 4,000 m-deep library. The 200 m deep ALOHA library, although having also a relatively similar temperature (18°C) to Km3 shares many features and genes with the photic region, and falls apart in neighbour-joining analyses (Fig. 7A). Therefore, in the absence of light, temperature, and not pressure, appears to be the major stratifying factor for microbial communities. Other factors might also have an effect, such as the limited transport of deep-ocean microbes to the Mediterranean through the Gibraltar sill. However, since identical o nearly identical sequences for conserved (16S rRNA gene) and, furthermore, variable (16S-23S rRNA intergenic spacer) markers have been retrieved from the deep Mediterranean and open oceans [8], [38], the colonization of the Mediterranean by deep oceanic microorganisms is possible. Therefore, temperature rather than other physic-chemical or biogeographical parameters seems the most influential stratification factor in these waters.

**Table 4:** ALOHA and Km3 oceanographic data.

	North Pacific ALOHA							Mediterranean Km3
coordinates	22°45′N, 158°W							36°30′N, 15°40′E
max.depth (m)	4800							3243
**depth (m)**	**10**	**70**	**130**	**200**	**500**	**770**	**4000**	**3010**
Sampling Time	Oct. 7^th^ 2002	Oct. 7^th^ 2002	Oct. 6^th^ 2002	Oct. 6^th^ 2002	Oct. 6^th^ 2002	Dec. 21^st^ 2003	Dec. 21^st^ 2003	Nov. 17^th^ 2004
Temp. (°C)	26.40 (24.83±1.27)*	24.93 (23.58±1.00)*	22.19 (21.37±0.96)*	18.53 (18.39±1.29)*	7.25 (7.22±0.44)*	4.78 (4.86±0.21)*	1.46 (1.46±0.01)*	13.93 (13.80±0.05)*
Salinity (PSU)	35.08 (35.05±0.21)*	35.21 (35.17±0.16)*	35.31 (35.20±0.10)*	35.04 (34.96±0.18)*	34.07 (34.06±0.03)*	34.32 (34.32±0.04)*	34.69 (34.69±0.00)*	38.74 (38.69±0.03)*
Chl (μg/Kg)	0.08 (0.08±0.03)*	0.18 (0.15±0.05)*	0.10 (0.15±0.06)*	0.02 (0.02±0.02)*	ND	ND	ND	ND
DOC (μM/Kg)	78 (90.6±14.3)*	79 (81.4±11.3)*	69 (75.2±9.1)*	63 (60.4±9.8)*	47 (47.8±6.3)*	39.9 (41.5±4.4)*	37.5 (42.3±4.9)*	54.2±5.85*
Oxygen (μM/Kg)	204.6 (209.3±4.5)*	217.4 (215.8±5.4)*	204.9 (206.6±6.2)*	198.8 (197.6±7.1)*	118.0 (120.5±18.3)*	32.2 (27.9±4.1)*	147.8 (147.8±1.3)*	203.7 (202.66±1.2)*
DIP (nmol/Kg)	41.0 (56.0±33.7)*	16 (43.1±25.1)*	66.2 (106.0±49.7)*	274.2±109.1*	2153 (2051±175.7)*	3070 (3000±47.1)*	2558 (2507±19)*	159.0±22.6*
N+N (nmol/Kg)	1.0 (2.6±3.7)*	1.3 (14.7±60.3)*	284.8 (282.9±270.2)*	1161.9±762.5*	28850 (28460±2210)*	41890 (40940±500)*	36560 (35970±290)*	4706±133.3*
SLCA (μl/Kg)	1.30±0.37*	1.34±0.37*	1.72±0.56*	5.31±0.74*	45.37±5.75*	92.06±4.08*	160*	8.32±0.24*
HPP (cell×10^4^ ml^−1^)	30.2±16.2*	25.2±9.9*	19.9±6.9*	13.03±2.5*	5.19±1.5*	3.15±0.7*	0.55±0.06*	3.1±1.73*
POC (μM C / Kg)	2.16±0.54*	1.97±0.35*	1.29±0.36*	0.55±0.15*	0.39±0.13*	0.30±0.13*	-	1.925±0.56*

Values shown are those from the same CTD casts as the samples (DeLong *et al.* 2006 and this work). *Archival data are from ALOHA HOT-DOGS© database (http://hahana.soest.hawaii.edu/hot/hot-dogs/) or in the case of Km3 from the ICES oceanographic database (http://www.ices.dk/ocean) and correspond to several datasets collected at the depth and approximate location (less than 50 NM away) as the samples. Values in parentheses are the average value±standard deviation. Abbreviations are Temp, Temperature; Chl, chlorophyll; DOC, dissolved organic carbon; DIP, dissolved inorganic phosphate; N+N, nitrate plus nitrite; SLCA, silicate; HPP, heterotrophic picoplankton (DAPI counts); POC, particulate organic carbon.

## Discussion

This is the second large sequencing effort carried out in the deep ocean, the only precedent being the central Pacific gyre water column study at the ALOHA station [14]. In our case a single depth (3,000 m) was sampled at very pristine waters in the Ionian Sea, and its study adds interesting complementary information to the previous work in two ways. First, it provides a second geographic location belonging to a very different water mass, not only distant from the Pacific sampling site but also differing in various fundamental parameters, notably temperature. Furthermore the Gibraltar sill also precludes deep ocean currents from reaching the Deep Med and would isolate this habitat from the input of psycrophiles from Antarctic waters which relatives seem to populate the global ocean bathypelagic regions [39]. Another notable difference of the deep Mediterranean (Table 4) is the lower concentrations of inorganic nutrients N and P (about an order of magnitude lower) and, contrastingly, a higher biomass density in the Mediterranean that seems to be at least as active as in the deep global ocean. Thus, these nutrients do not seem to be limiting. Actually archival data of biomass estimators, like temperature, are more similar to the mesopelagic global ocean than the deep. Hence, common observations in deep Mediterranean and Pacific metagenomes may reasonably be taken as general deep ocean traits.

We found that the assignment of fosmid-ends to already sequenced microbial genomes, something similar to the “genome fragment recruitment” used recently by Rusch et al. to analyze metagenomic libraries from the Global Ocean Sampling transect [13], was very useful to analyze our data and predict microbial lifestyles. However, these analyses are to be taken cautiously as many of our sequences did not match known genes or could not be confidently assigned to defined taxa (Table 1). Certainly, many more marine genomes covering the whole phylogenetic spectrum would be needed to have a picture of better resolution. Yet from the taxa and gene functional categories identified in this way, some conclusions can be drawn about the lifestyle and ecosystem functioning in the deep Mediterranean. Except for the crenarchaeota, most of the remaining lineages in deep Mediterranean waters are likely heterotrophs, as confirmed by the classification of gene functions and metabolic pathways (Table 1, Figs. 5 and 6), including transporters, particularly for amino acids and carboxylic acids, and catabolic routes involved in complex organic degradation, e.g. xenobiotics. This is in agreement with an essential role of the heterotrophic deep-sea microbes in the mineralization of organic carbon [40], [41]. In many ways, they are to the marine phytoplankton what the soil microbiota are to the forest. The presence of microbial groups typically found in soil (Rhizobiales, Actinobacteria, Acidobacteria) would indeed be consistent with the ecological role of the deep ocean as an “invisible soil” for the “invisible forest” [40]. Our own analysis show that these groups appear also at the ALOHA aphotic zone samples (Figs. 2 and 3). These lineages might live attached to sinking particles. The presence of planctomycetes, often associated to sinking particles [42], also points in this direction. In most marine metagenomic studies carried out do date, particles were excluded by restrictive pre-filtration (0.8 µm), partly with the objective of excluding eukaryotic (including picoeukaryotic) cells [12], [13]. As we used 5 µm pore-sized filters for the pre-filtration step, it might be possible that we captured more biomass from marine snow particles.

At any rate, our vision of the deep ocean ecosystem functioning is changing. Not only chemolithoautotrophy (crenarchaeota) appears to be significant, but also mixed strategies including lithoheterotrophy may be as important as pure heterotrophy. Thus, we detected a considerable number of *cox* genes encoding different subunits of the carbon monoxide dehydrogenase, CODH, responsible for the aerobic oxidation of CO. Initially thought to be exclusive of autotrophs, CO oxidation is being discovered in a plethora of organisms, including members of the marine *Roseobacter* clade [23], [36], [37]. Though present in photic layers, CODH genes are more abundant in deep layers of the ALOHA water column [14]. This strongly suggests that deep-sea microorganisms oxidize carbon monoxide (and perhaps other reduced substrates) released from tectonically active areas or anaerobic microenvironments, as alternative or complementary energy sources to heterotrophy. This energy metabolism versatility would be advantageous in this highly depleted environment, where secondary production might be boosted by chemolithotrophy much in the way that phototrophy helps heterotrophy at the surface [43].

The comparison of our deep Mediterranean data with those obtained in the Pacific ALOHA water column suggests that, in the absence of light, temperature becomes the major stratifying factor for community structure (Fig. 7). This effect of temperature seen with depth corroborates analogous temperature-dependent patterns in surface waters in the recent GOS metagenomic study [13] or some cases of sharp ecotype differentiation [38], [44] In this sense, temperature might be the second most relevant environmental factor operating in the global open ocean, the first being accessibility to light as energy source, while pressure would seem less critical in determining community structure and lifestyles in the deep ocean, at least down to about 4,000 m. This does not imply that piezophilic microbes are not important but are probably much more relevant in very deep trenches. By contrast, temperature is a crucial parameter that requires specific long-studied molecular adaptations [21], [45], [46]. Low temperatures in most bathypelagic habitats prevent the metabolism of many microbes beyond a certain threshold. By contrast, in the Mediterranean, the persistence of warm temperatures down to bathypelagic waters would allow the persistence of mesopelagic microbial communities adapted to aphotic regions but unable to cope with near-zero temperatures where more psychrophilic organisms dominate.

## Methods

### Sample collection

250 l of seawater were collected by using Niskin bottles mounted on a General Oceanics rosette from a depth of 3,010 m (sea-bottom at 3,243 m depth) at the Ionian Km3 station (36°29′98″N, 15°39′97″E) in November 17^th^ 2004 during a cruise of the R/V Urania. Water temperature was 13.93°C and salinity 38.75 PSU. Seawater was sequentially filtered through a 5 µm pore size polycarbonate filter and the filtrate passed through 0.22 µm pore size Sterivex filters (Durapore, Millipore) using a peristaltic pumping system. Sterivex filters were filled with lysis buffer (40 mM EDTA, 50 mM Tris/ HCl, 0.75M sucrose) and stored at −20°C, until DNA extraction.

### DNA extraction and fosmid library construction

Sample filters were thawed on ice and then treated with 1mg/ml lysozyme and 0.21 mg/ml proteinase K (final concentrations). Nucleic acids were extracted with phenol-chloroform-isoamyl alcohol (25∶24∶1) and chloroform-isoamyl alcohol (24∶1), and then concentrated with sterile water using a microconcentrator (Centricon 100, Amicon). DNA integrity was checked by agarose gel electrophoresis. A fosmid genomic library was constructed from approximately 1.1 µg of DNA from the 0.2–5 µm plankton fraction using the CopyControl™ Fosmid Library Production Kit (Epicentre) as described by the manufacturer's instructions. A total of 20,767 fosmid clones were obtained, which corresponds to *ca.* 725–830 Mpb environmental DNA assuming an average insert size of 35–40 Kbp.

### Screening and fosmid-end sequencing

The library was pooled in groups of 96 clones. DNA from pooled cultures was extracted using the QIAprep Spin Miniprep Kit (Qiagen) and then PCR-screened for the presence of archaeal and bacterial 16S rRNA genes. Different primer combinations were used for archaeal 16S rRNA gene amplification using 21F (5′-TTCCGGTTGATCCTGCCGGA), Ar109 (5′-AC(G/T)GCTGCTCAGTAACACGT), ANMEF (5′-GGCTCAGTAACACGTGGA) and 1492R (5′-GGTTACCTTGTTACGACTT). In the case of bacteria, we amplified 16S rRNA gene together with the adjacent intergenic spacer (ITS) using 27F (5′-AGAGTTTGATCCTGGCTCAG) and 23S1R (5′-GGGTTTCCCCATTCGGAAATC). In this way, only bacterial fosmids containing ITSs of different size to that of *Escherichia coli* were detected. PCR reactions were carried out under the following standard conditions: 35 cycles (denaturation at 94°C for 15 s, annealing at 50°C for 30 s, extension at 72°C for 2 min) preceded by 2 min denaturation at 94°C and followed by 7 min extension at 72°C. Twenty-eight 16S rRNA gene-containing archaeal clones and sixteen bacterial clones were detected in the library. The genes were sequenced (Genome Express, Meylan, France) and the closest relatives in databases searched using BLAST [47]. In parallel, the insert terminal sequences of *ca.* 5,000 fosmid clones were sequenced at the Göttingen Genomics Laboratory, Germany (http://www.g2l.bio.uni-goettingen.de). A total of 9,048 high quality sequence reads were obtained (average length 794 bp), which implies approximately 7.2 Mbp sequence, i.e. roughly two prokaryotic genome equivalents.

### Fosmid-end sequence analysis

Fosmid end sequences were revised and cleaned of vector contaminant sequences using Sequencher 4.1.4 software (Gene Codes Corp.). For taxonomic binning, sequences were queried against the NCBI non-redundant (nr) protein database using BLASTX using a cut-off value of <1e-50. Top BLAST high-scoring pairs (HSPs) were tabulated according to the NCBI taxonomic identifier for each sequence. For COG assignments, sequences were compared to the cluster of orthologous genes (COG) databases using BLAST (rpsblast (-p F)) using a cut-off value of 1e-7. Also, sequences were compared to the KEGG database using BLASTX. Results were tabulated, and used to determine the proportion of sequences contained in each COG category or KEGG pathway. To calculate the amino acid usage pattern, ORFs for each data sample were identified using the automated genome annotation software Glimmer 2.02 [48]. The frequency of each amino acid was represented as suggested by [49].

### Comparative analysis of marine metagenomic libraries

In order to compare the Km3 library with that of the Sargasso Sea [12] and the North-Pacific Subtropical Gyre ALOHA station [14], coverage plots were generated by using the Promer program implemented in MUMmer 3.18, using the “maxmatch” option [50] and visualized using the MUMmer-plot program (http://mummer.sourceforge.net/). For sequence analysis, resulting delta files were converted into coordinate files and sequence analysis by using the ‘show-coords’ option. To estimate cumulative protein sequence differences in Km3 and the water column at the ALOHA station, we made TBLASTX searches of the complete set of sequences from every single library versus all the others. The bitscores of the top HSPs from every single sequence from one set versus another were summed to yield a cumulative pairwise bitscore value that was normalized and used to construct a distance matrix. The matrix was analyzed using PAST software (v. 1.58) (http://folk.uio.no/ahoammer/past) by cluster analysis. Node support was assessed by Jackknife matrix resampling [51]. Cumulative pairwise bitscore values were normalized by dividing each one by the cumulative bistscore value derived from the TBLASTX of one dataset versus itself and the number of HSPs of each case.

### Phylogenetic analyses

Archaeal 16S rRNA gene sequences detected in Km3 fosmids were aligned using ClustalX [52] with those from the ALOHA water column and those from available Group I crenarchaeotal genomes and selected Group I and II archaeal genome fragments. We then made a preliminary neighbour-joining tree with the 244 used sequences in order to make a selection of representative sequences to be included in a maximum likelihood tree. In the case of bacteria, we included the closest relatives to the identified Km3 sequences by BLAST, as well as representative members of the detected bacterial phyla. Sequences were aligned using Clustal X, and the alignment manually edited using the ED program of the MUST package [53]. Gaps and ambiguously aligned positions were excluded from our analyses. Maximum likelihood trees were reconstructed using PhyML [54] applying a general time reversible model of sequence evolution (GTR), and taking among-site rate variation into account by using a six-category discrete approximation of a distribution and a proportion of invariable sites. ML bootstrap proportions were inferred using 1000 replicates. For phylogenetic analysis of the Cox proteins, TBLASTX searches were carried out, the corresponding sequences recovered from GenBank and a multiple alignment generated using Clustal X and manually refined as mentioned above. Maximum likelihood trees were reconstructed using PhyML [54] with the JTT model of sequence evolution and taking among-site rate variation into account by using a six-category discrete approximation of a distribution and a proportion of invariable sites. ML bootstrap proportions were inferred using 1000 replicates. Cox phylogenetic trees are available upon request. Phylogenetic trees were viewed using the program TREEVIEW [55].
